# Aspirin inhibited the metastasis of colon cancer cells by inhibiting the expression of toll-like receptor 4

**DOI:** 10.1186/s13578-018-0234-2

**Published:** 2018-05-21

**Authors:** Suaka Kagbo-Kue, Taiwo Ajose, Nicolas Bakinde

**Affiliations:** Department of Internal Medicine, Morehouse School of Medicine, Atlanta, USA

Dear Editor,

It was interesting to read the article *Aspirin inhibited the metastasis of colon cancer cells by inhibiting the expression of toll*-*like receptor 4* published by Ying et al. Several studies have explored the mechanism of Aspirin in preventing colorectal cancer (CRC) and also that of metastases in CRC [1–4] but this study was noteworthy in that it explored the mechanism of Aspirin in preventing liver metastases in CRC.

According to the National Cancer Institute, CRC is the second leading cause of cancer deaths in the United States [5]. The liver is the most common site of metastases in CRC and significantly affects survival [6, 7].

Aspirin is chemopreventive in different types of cancers especially CRC [1]. Tumorigenesis and CRC metastasis are hypothesized to occur by different mechanisms involving cancer stems cells (CSC), the overexpression of cyclooxygenase-2 (COX-2) and epidermal growth factor (EGFR), and other downstream effects in the early phases of CRC [2–4]; Aspirin is postulated to play its role by modifying these epigenetic events, especially among patients with mutated-PIK3CA [4]. Aspirin taken chronically at doses ranging from 75 to 200 mg is associated with reduced mortality and increased survival [3, 4].

The results reported by the authors demonstrate that lipopolysaccharide (LPS) induced the metastasis and epithelial-mesenchymal transition (EMT) phenotype of colon cancer cells via a toll-like receptor 4 (TLR4) dependent manner. TLR4 thus mediates the biologic function of LPS. Previous research has also indicated that LPS could activate the nuclear factor kappa B (NFkB) signal transduction which also plays a role in the EMT process [8]. LPS thus potentiated the metastatic potential of colon cancer cells in the cell lines in this study (C26 and HCT116). Treatment with Aspirin decreased TLR4 expression by decreasing LPS-induced EMT which further leads to downregulation of NFkB [7].

In agreement with the findings from the study, Aspirin might act as an inhibitor in the LPS-induced metastasis of colon cancer and TLR4 could be used as a prognostic marker and a potential therapeutic target [7]. Another class of drugs to consider is Statins. Statins have also been shown to inhibit LPS-induced EMT via the downregulation of TLR4 and NFkB in human biliary epithelial cells, and could be an additional agent for reducing the metastatic potential of colon cancer cells [9].

Based on this study, Aspirin is an inexpensive drug that could potentially reduce the mortality of CRC. Further studies are needed to explore the application to independent cohorts of patients, to validate that the potential benefits outweigh the possible risks, and to determine the role of other inexpensive drugs such as Statins, so as to increase survival in CRC.
